# Combination olaparib and cyclophosphamide cancer therapy exacerbates ovarian reserve depletion in mice with conditional loss of *Brca1* in oocytes

**DOI:** 10.3389/fendo.2026.1885969

**Published:** 2026-08-12

**Authors:** Yujie Cao, Zaahida Abdul Jalil, Xinyi Mu, Jessica M Stringer, Nadeen Zerafa, Amy L. Winship, Karla J. Hutt

**Affiliations:** Women’s Health and Ovarian Biology Laboratory, Biomedicine Discovery Institute, Department of Anatomy and Developmental Biology, Development and Stem Cells Program, Monash University, Clayton, VIC, Australia

**Keywords:** BRCA1 mutation, cancer therapy, female fertility, *in vitro* fertilization, ovarian reserve

## Abstract

**Introduction:**

Olaparib, a small-molecule poly(ADP-ribose) polymerase (PARP) inhibitor, selectively impairs single-strand DNA repair and improves outcomes in patients with *BRCA1*-mutant cancers by promoting the accumulation of unrepaired DNA damage in tumor cells. It is increasingly used in combination with chemotherapy to enhance therapeutic efficacy and overcome resistance; however, its impact on ovarian function in *BRCA1* mutation carriers remains unknown. Here, we investigated whether BRCA1 deficiency alters ovarian sensitivity to combined cyclophosphamide and olaparib exposure.

**Methods:**

Adult wild-type (WT; *Brca1*^fl/fl^*Gdf9*^+/+^) and oocyte-specific *Brca1* conditional knockout (cKO; *Brca1*^fl/fl^*Gdf9*^cre/+^) mice were treated with cyclophosphamide (75 mg/kg, single intraperitoneal injection) or vehicle, followed by daily olaparib (50 mg/kg, subcutaneous) or vehicle for 28 days.

**Results:**

Ovarian reserve, endocrine function, estrous cyclicity, and IVF outcomes were assessed. Combination treatment did not affect serum AMH levels, estrous cyclicity, or IVF outcomes in either genotype, indicating no evidence of short-term impairment of reproductive function. Importantly though, *Brca1* cKO mice exposed to combination treatment exhibited long-term effects, evident from a significant depletion of primordial follicles compared with genotype-matched vehicle controls, whereas ovarian reserve remained preserved in WT animals receiving the same treatment regimen.

**Discussion:**

These findings demonstrate that BRCA1 deficiency may confer selective susceptibility of the ovarian reserve to combination chemotherapy and PARP inhibitor exposure without immediate impairment of endocrine or reproductive function. This study provides the first evidence that *BRCA1* mutation carriers may be uniquely vulnerable to accelerated primordial follicle loss following chemotherapy PARP inhibitor combination therapy, with important implications for fertility preservation and long-term reproductive health in young women undergoing cancer treatment.

## Introduction

The ovarian reserve is established prior to birth and consists of a finite pool of non-renewable primordial follicles containing oocytes arrested in meiosis and maintained in a state of growth arrest. Upon activation, primordial follicles develop through the process of folliculogenesis, to be ovulated as a mature oocyte, or undergo degeneration, known as follicle atresia (1). Follicle loss is a physiological process, although depletion can be accelerated by exogenous insults, like cancer treatments.

In recent decades, improvements in cancer detection and therapy have increased overall cancer survivorship. However, since many treatments induce gonadal toxicity, examining the impacts of cancer therapies on survivors’ reproductive function has emerged as a research priority. Some studies report that more than 50% female cancer survivors face premature ovarian insufficiency (POI), and 38% could experience pregnancy failure after receiving cancer treatment (2, 3). The American Society of Clinical Oncology guidelines characterize fertility preservation as an essential consideration for patients prior to and after cancer treatment (4). In young women, cancer treatment-induced ovarian damage can lead to temporary effects, or permanent damage depending on the class of ovarian follicles targeted by treatment and the extent of follicle depletion (5, 6).

Cyclophosphamide is among the most widely used chemotherapeutic agents for cancers affecting women of reproductive age, including breast cancer, lymphomas, and leukemias (7, 8). As a prodrug alkylating agent, it requires hepatic activation by cytochrome P450 enzymes to generate its cytotoxic metabolites, phosphoramide mustard and acrolein (9). Phosphoramide mustard exerts antineoplastic activity by forming covalent DNA interstrand and intrastrand crosslinks, thereby disrupting DNA replication and transcription (10). Cyclophosphamide-induced ovotoxicity is well established and it is classified among the most highly gonadotoxic chemotherapies, causing primordial follicle depletion, granulosa cell apoptosis, and increased oxidative stress within the ovary (11).

According to the International Agency for Research on Cancer, breast cancer is one of the most commonly diagnosed malignancies worldwide (12). It accounts for approximately 11.6% of all new cancer cases globally and is a leading cause of cancer-related mortality in women, representing 6.9% of global cancer deaths (12). Breast cancer incidence is associated with both sporadic and hereditary factors, including germline mutations in the breast cancer susceptibility genes (*BRCA)1* and *2* (13). BRCA1/2 are essential for the detection and repair of DNA double-strand breaks via the error-free homologous recombination pathway (14). Individuals carrying germline *BRCA1/2* mutations have an estimated lifetime risk of up to 72% for breast cancer and 15% for ovarian cancer compared with non-carriers (15). Consequently, many carriers will require cancer treatment, highlighting the importance of understanding treatment effects on ovarian function in this population. Notably, emerging evidence suggests impaired reproductive function in *BRCA1* mutation carriers compared with the general population (reviewed in (16)). Mechanistically, loss of BRCA1 function compromises oocyte DNA repair capacity, leading to increased genomic instability and accelerated depletion of the ovarian reserve in both clinical and preclinical studies (17–19).

Poly(ADP-ribose) polymerase 1 (PARP1) is another key regulator of the DNA damage response, acting as an early sensor of DNA single-strand breaks and facilitating their repair. In contrast to BRCA1, which mediates repair of DNA double-strand breaks via homologous recombination, PARP1 is primarily involved in base excision repair (BER), the pathway responsible for resolving single-strand DNA lesions (20, 21). Given its central role in DNA repair, pharmacological inhibition of PARP1 by PARP inhibitors, such as olaparib, leads to the persistence of unrepaired single-strand breaks, which are converted into double-strand breaks during DNA replication. This ultimately results in cell death through synthetic lethality (22, 23). Olaparib selectively induces apoptosis in BRCA-deficient cells (24) and has demonstrated improved response rates in patients with treatment-resistant cancers (25).

Chemotherapy is still one of the main strategies for treating cancers, although low responses are often identified due to treatment resistance (26, 27). To overcome this, combination therapy with olaparib is now often preferred to improve treatment outcomes (28, 29). In this study, we evaluated the impacts of combination cyclophosphamide and olaparib treatment on ovarian function in the context of loss of *Brca1* in oocytes in mice. We hypothesized that oocyte-specific loss of *Brca1* may exacerbate combination therapy-induced ovarian damage.

## Materials and methods

### Animals and ethics statement

Animals involved in this study were maintained on a colony with a crossed background (FVB/N x C57BL6/J). The conditional knock-out was generated as previously described (19). This study used two genotypes, WT (*Brca1*^fl/fl^*Gdf9*^+/+^) and the *Brca1* cKO (*Brca1*^fl/fl^*Gdf9*^cre/+^). Adult WT F1 (C57BL6/J x Balb/cJ) male mice were obtained from the Monash University Animal Research Platform (MARP). All animals were housed under a 12hour light-to-dark cycle, temperature-controlled and high barrier facility. All animals were free to water and food accesses. All animal procedures were performed in accordance with the National Health and Medical Research Council Australian Code for Practice for the Care and Use of Animal in Science and all experiments were approved by the MARP Animal Ethics Committee (MARP4 #34047).

### Combination treatment with cyclophosphamide and olaparib

On day 1, adult postnatal day (PN)40–80 WT and *Brca1* cKO mice at peak fertility were weighed and received a single dose of cyclophosphamide (75 mg/kg/body weight; Sigma-Aldrich #C0768-5G, Merck KGaA, Darmstadt, Germany) or vehicle control (saline; 100 µL) by intraperitoneal injection. From day 2 onwards, animals received daily subcutaneous injection of olaparib (50 mg/kg/body weight; MedChemExpress, #HY-10162-1G, NJ, USA) in 10% DMSO (Sigma) and saline containing 10% w/v 2-hydroxypropyl-β-cyclodextrin (Sigma), or saline vehicle control containing 10% DMSO and 10% w/v 2-hydroxypropyl-β-cyclodextrin for 28 days. Body weight, assessed at the end of the experiment, did not differ significantly between treatment or genotype groups (**Supplementary Figure 1****).**

### Estrous cycle analysis

During treatment days 15 to day 28, 14 consecutive days of vaginal smears were performed on animals. Slides containing cells from the smears were stained via Rapid Diff stain kit (Australian Biostain, #029-ARD.K, SA, Australia) and assessed using the Leica DM500 light microscope (Lecia). Smears were categorized into four different stages (proestrus, estrus, metestrus and diestrus) based on the cell types dominantly present as previously described (30). The number of days of animals spent at each stage were tracked and quantified.

### *In vitro* fertilisation (IVF)

Both *Brca1* cKO and WT females underwent superovulation 24h post final treatment (d29 + 24 hours) for IVF. All animals were primed with 10 international units (IU, intraperitoneal injection) of pregnant mare serum gonadotrophin (PMSG; Intervet, Merck & Co, NJ, USA) to optimize the number of matured oocytes. Then 48h later, 10 IU of human chorionic gonadotrophin (hCG, intraperitoneal injection; Intervet) was administered to trigger ovulation. Animals were humanely sacrificed by isoflurane inhalation, followed by terminal cardiac puncture for the collection of oviducts to harvest cumulus-oocyte-complex and ovaries 16h post-hCG. On the collection day for IVF, adult F1 (C57BL6/J x Balb/cJ) male mice were humanely killed to obtain sperm. Epididymis tissue was collected and punctured to release the sperm. The punctured epididymis was immersed and cultured in the G-IVF PLUS (Vitrolife, Gothenburg, Sweden) media for minimum 30 mins at 37 °C to allow the sperm to swim up. For insemination, surface layer of the sperm swim-up were co-cultured for 4–6 hours with cumulus-oocyte-complex (10µL per COC). Oocytes were transferred into the culture media (#67010060A, SAGE 1-Step, Måløv, Denmak) for a 4-day incubation after being inseminated. A media change was performed 1-day post insemination. Fertilization was determined by the number of 2-cell embryos formed at 1-day post insemination. Fertilization rate was calculated based on the number of 2-cell embryos formed from the number of healthy oocytes collected. Blastocyst rate was determined by the number of blastocysts formed from the number of healthy 2-cell formed.

### Follicle quantification

One ovary was harvested from each animal for follicle quantification. On the collection day, ovaries were fixed in 10% (vol/vol) neutral buffered formalin (10%) solution for 24 hours and paraffin embedded. Ovaries were exhaustively sectioned at a thickness of 5µm. Every ninth tissue section was collected and stained with periodic acid-Schiff and hematoxylin. Tissue sections were scanned using the Aperio Digital Pathology Slide Scanner (Leica Biosystems, 20x objective) and images assessed by one blinded assessor on the Aperio ImageScope (Leica Biosystems). The total number of healthy primordial, primary, unhealthy primordial and primary was quantified as previously detailed (31, 32). Due to the hormone priming for superovulation, developing follicles (secondary and antral) were not quantified in this cohort. Follicles were only counted when a clear oocyte nucleus was present to avoid double counting. The total follicle number was estimated with multiplying the raw counts by 9 (the uncounted sections).

### Serum anti-Müllerian hormone

Peripheral blood was obtained via cardiac puncture at collection and then centrifuged at 500g for 5 minutes at room temperature. Serum was collected and stored at -80 °C. The AMH ELISA kit (#AL-113, Ansh Labs, Texas, USA) was used to quantify AMH levels. The ClarioStar microplate reader (BMG Labtech) was used to measure the absorbance for quantification.

### Statistical analysis

Data were compared between treatment in the same genotype, as well as between WT and *Brca1* cKO animals that received the same treatment regimens. All statistical analysis was performed via GraphPad Prism 9 software (GraphPad Software, Inc., La Jolla, CA, USA). Data are presented as mean ± SEM. The Shapiro-Wilk test was used to determine the normality of each data set. For normally distributed data, one-way ANOVA with Holm-Šídák *post-hoc* test was used to compare the mean between more than two groups. Whereas a Kruskal-Wallis test with Dunn’s multiple comparison *post-hoc* test was used to when the data set was non-normally distributed. The Spearman’s rank test was applied for analyzing a potential correlation between serum AMH level and primordial follicle numbers. The statistical significance was set at p<0.05.

## Results

### Combination cyclophosphamide and olaparib treatment did not alter estrous cyclicity in WT and *Brca1* cKO mice

**Figure 1:**
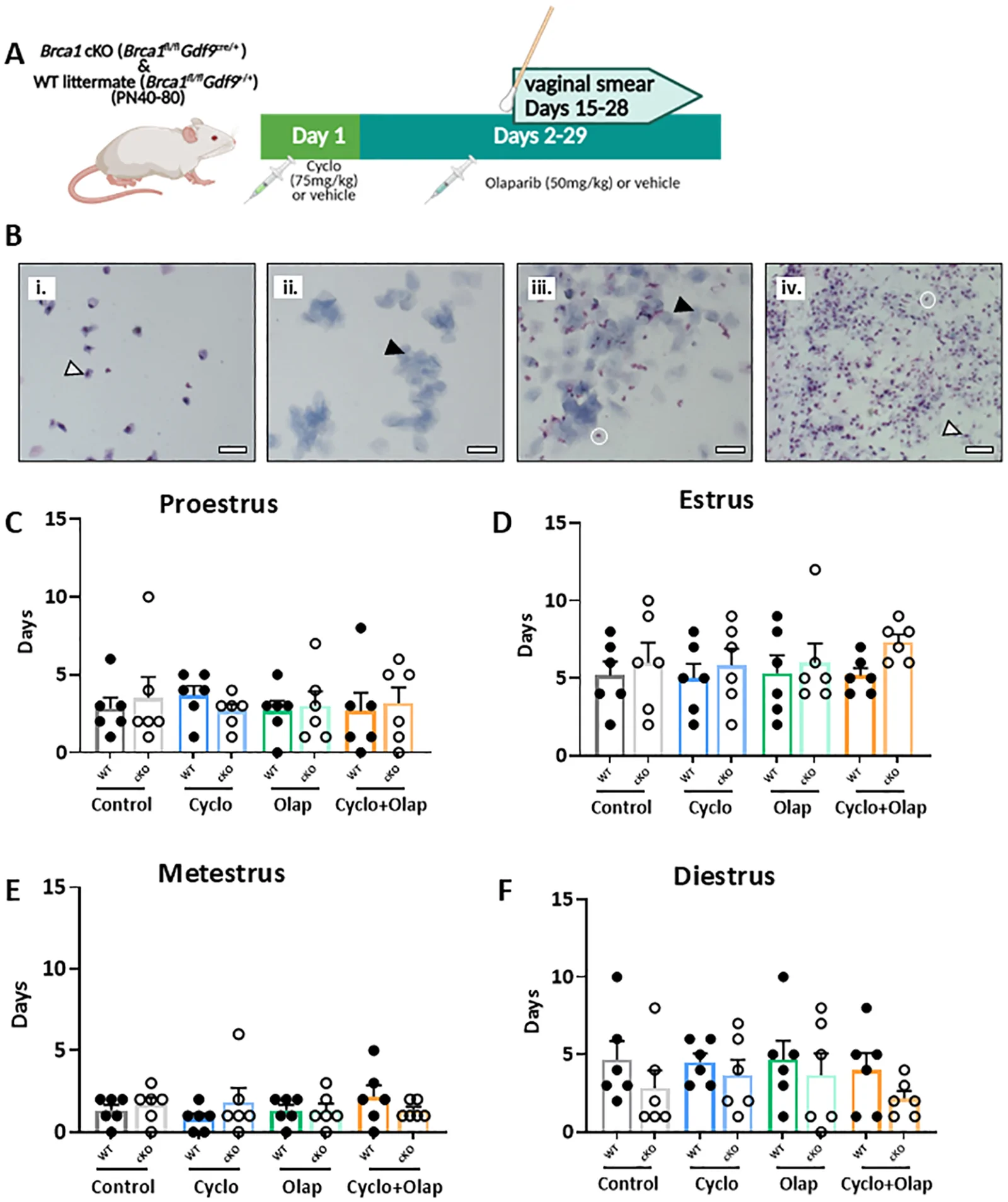
The effects of combination treatment of cyclophosphamide and olaparib on mouse estrous cycling in WT and Brca1 cKO mice. The schematic shows combination therapy of cyclophosphamide + olaparib, followed by vaginal smears to assess estrous cycling in WT and cKO animals at PN40-80 **(A)**. Representative images of 4 distinct estrous stages: Proestrus (Bi), Estrus (Bii), Metestrus (Biii) and Diestrus (Biv). Nucleated epithelial cells (white arrowhead) are the dominant cell type during proestrus and are gradually replaced by large, cornified epithelial cells (black arrowhead), which are the only cells present during estrus. The appearance of leukocytes (white circle) indicates metestrus, and their numbers increase in diestrus alongside the reappearance of nucleated epithelial cells. Scale bar = 50 µm **(B)**. Number of days animals spent in each phase of the cycle including proestrus **(C)**, estrus **(D)**, metestrus **(E)** and diestrus **(F)**. Each data point represents one animal (Control WT n=6; Control cKO n=6; Cyclo WT n=6; Cyclo cKO n=6; Olap WT n=6; Olap cKO n=6; Cyclo+Olap WT n=6; Cyclo+Olap cKO n=6);. Data are presented as mean ± SEM. One-way ANOVA with Holm-Šídák *post-hoc* test for parametric data, Kruskal-Wallis test with Dunn’s multiple comparison *post-hoc* test for non-parametric data. Data are considered significant at *p* < 0.05.

Adult WT and oocyte-specific *Brca1* cKO female mice received a single dose of cyclophosphamide or vehicle control, followed by olaparib or vehicle administration for 28 days to model combined chemotherapy and PARP inhibitor exposure. The dose of cyclophosphamide was deliberately chosen so as not to dramatically deplete primordial follicles and to investigate potential synergistic impacts with olaparib. To assess estrous cyclicity during treatment, vaginal cytology was performed daily from treatment days 15–28 (**Figure 1A**). The murine estrous cycle is analogous to the human menstrual cycle and is tightly regulated by ovarian hormone production. It comprises four distinct stages, each defined by characteristic dominant cell populations identified by vaginal cytology (**Figure 1B**). Estrous cycles typically range from 2–8 days in length, most commonly 4–5 days (30–32). Comparison of the total time spent in each estrous stage over the 14-day assessment period revealed no significant differences between genotypes or treatment groups (**Figures 1C–F**), indicating that estrous cyclicity was not disrupted by BRCA1 deficiency or chemotherapy and PARP inhibitor treatment.

### Combination cyclophosphamide and olaparib treatment did not affect *in vitro* fertilization (IVF) outcomes in WT and *Brca1* cKO animals

**Figure 2:**
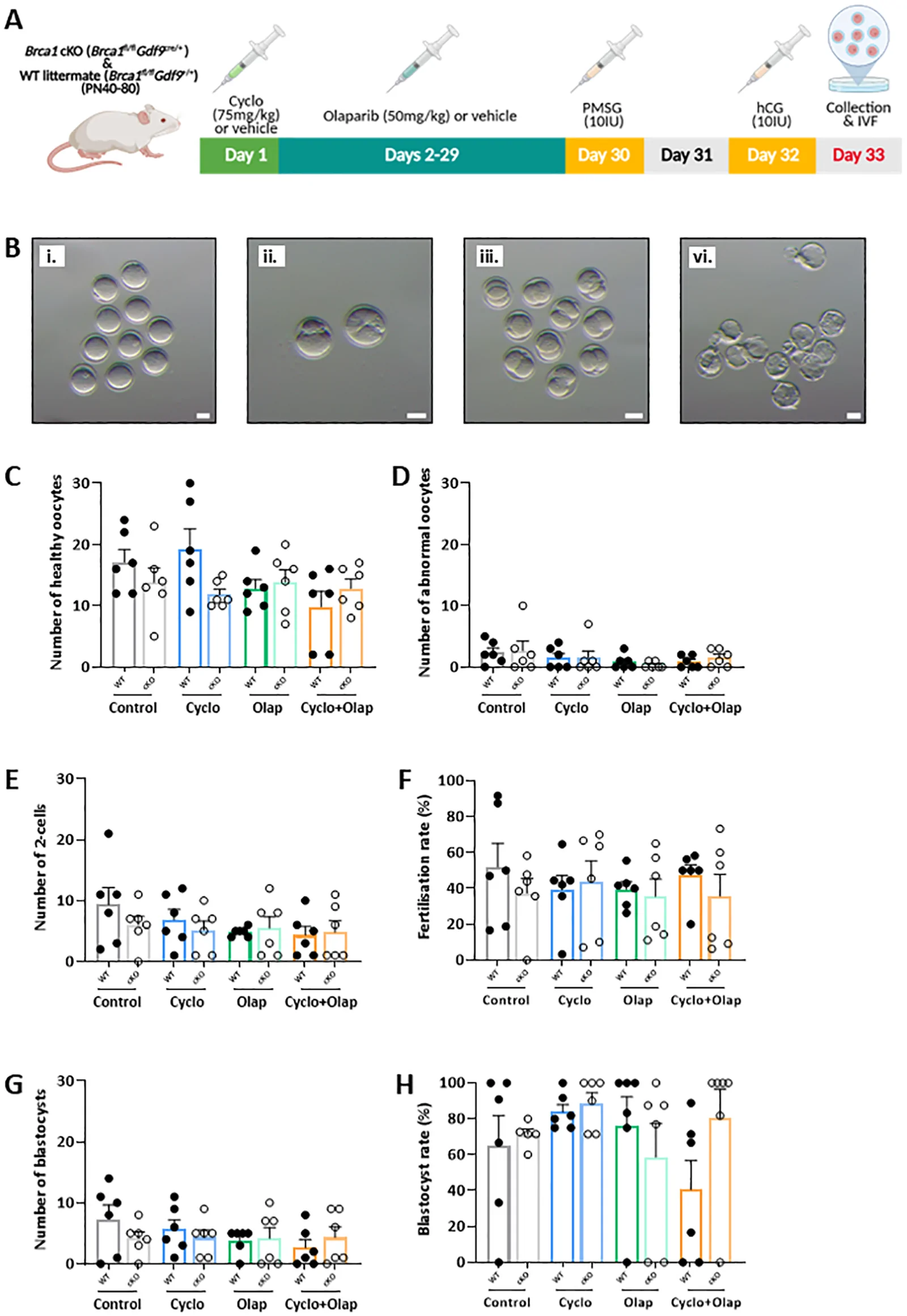
Impacts of combination treatment of cyclophosphamide and olaparib on IVF outcomes in PN40–80 WT and Brca1 cKO animals. Schematics of the treatment timeline **(A)**. Representative images of healthy **(Bi)** and fragmented/abnormal (Bii) oocytes 4–6 hours post insemination, healthy fertilised 2-cell embryos 1-day post insemination (Biii), healthy expanded hatching blastocysts 4-day post insemination (Biv). Scale bar = 50 µm **(B)**. Number of healthy oocytes **(C)**, abnormal oocytes **(D)**, 2-cells **(E)** and blastocysts **(G)**. Rate of fertilization (#2-cell/#healthy oocyte, **F**) and blastocysts (#balstocysts/#2-cell, **H**). Each data point represents one animal (Control WT n=6; Control cKO n=6; Cyclo WT n=6; Cyclo cKO n=6; Olap WT n=6; Olap cKO n=6; Cyclo+Olap WT n=6; Cyclo+Olap cKO n=6);. Data are presented as mean ± SEM. One-way ANOVA with Holm-Šídák *post-hoc* test for parametric data, Kruskal-Wallis test with Dunn’s multiple comparison *post-hoc* test for non-parametric data. Significance is set at *p* < 0.05.

WT and *Brca1* cKO mice were treated with vehicle, cyclophosphamide, olaparib, or the combination, followed by controlled ovarian stimulation and IVF to assess ovulatory function and the developmental competence of retrieved oocytes (**Figure 2A**). Oocytes were classified as morphologically healthy or abnormal, and subsequent fertilization and embryo development were assessed *in vitro*. Key outcomes included the number of oocytes retrieved, fertilization efficiency, 2-cell embryo formation, and blastocyst development (expanded blastocoel cavity formation). Across all measures, no significant differences were observed between treatment groups or genotypes (**Figures 2B–H**).

### Combination cyclophosphamide and olaparib treatment decreased primordial follicle numbers in *Brca1* cKO mice

**Figure 3:**
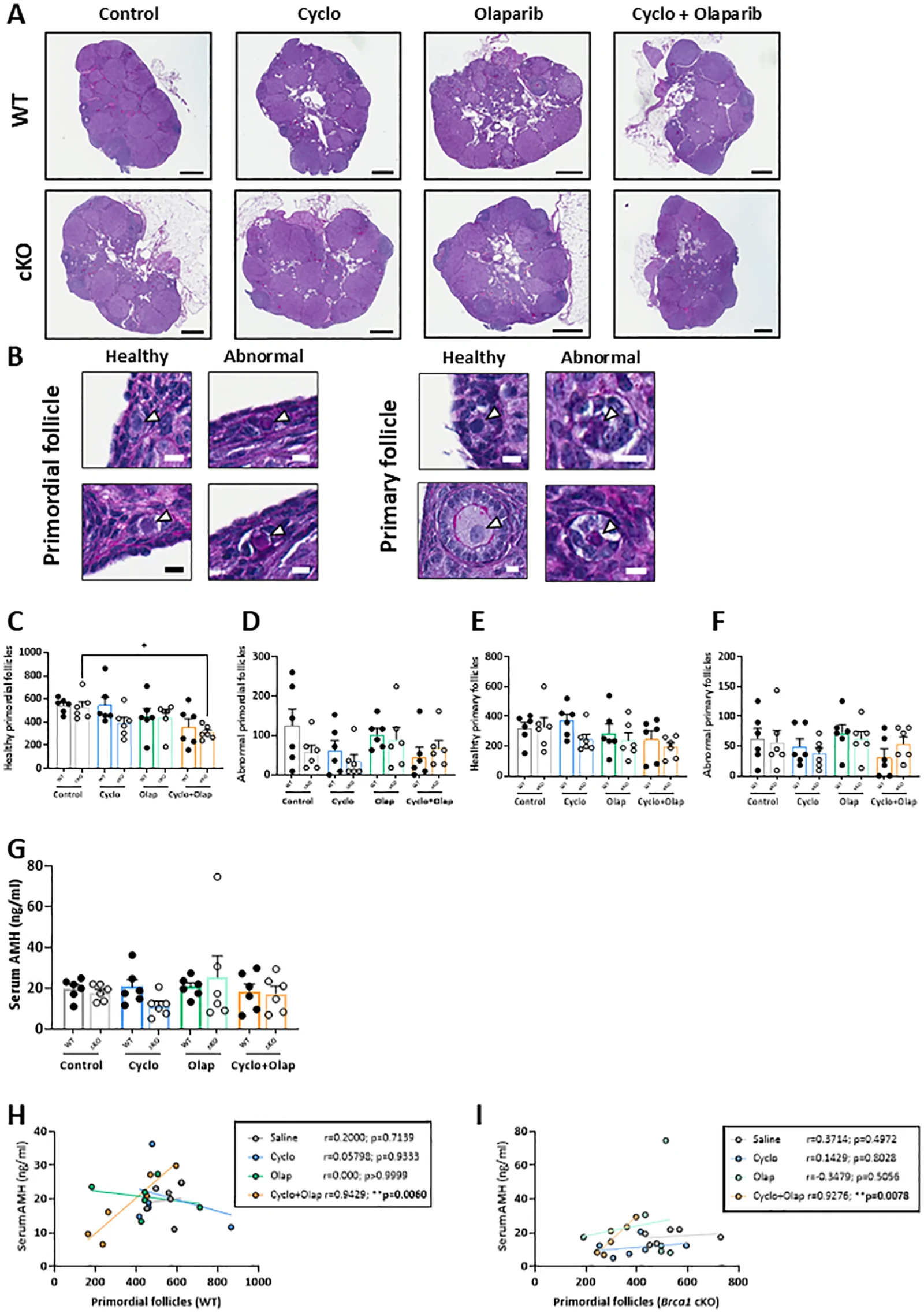
The impact of combination treatment of cyclophosphamide and olaparib on ovarian follicles in WT and *Brca1* cKO mice. Representative images of PAS-stained ovarian tissue of PN40–80 WT and *Brca1* cKO mice post either monotherapy or combination treatment, scale bar = 500 µm **(A)**. Representative images of PAS stained healthy and abnormal primordial and primary follicles, scale bar = 10 µm **(B)**. Number of healthy **(C)** and abnormal **(D)** primordial follicles. Number of healthy **(E)** and abnormal **(F)** primary follicles. Serum AMH levels (ng/ml) in WT and *Brca1* cKO **(G)**. Correlation of serum AMH levels (ng/ml) to primordial follicle numbers for both WT **(H)** and *Brca1* cKO **(I)**. Each data point represents one animal (Control WT n=6; Control cKO n=6; Cyclo WT n=6; Cyclo cKO n=6; Olap WT n=6; Olap cKO n=6; Cyclo+Olap WT n=6; Cyclo+Olap cKO n=6);. Data are presented as mean ± SEM. One-way ANOVA with Holm-Šídák *post-hoc* test for parametric data, Kruskal-Wallis test with Dunn’s multiple comparison *post-hoc* test for non-parametric data. The correlation was determined by Spearman’s rank. Significance is set at *p* < 0.05. *p = 0.0456 for primordial follicle count comparison; **p = 0.0060 for primordial follicle and serum AMH correlation in WT mice treated with combination olaparib cyclophosphamide therapy; **p = 0.0078 for primordial follicle and serum AMH correlation in cKO mice treated with combination olaparib cyclophosphamide therapy.

Primordial and primary follicles were quantified in ovarian tissue sections and classified as either healthy or abnormal (**Figures 3A, B**). Later-stage follicles were not evaluated as the animals had undergone a stimulation cycle immediately prior to ovary collection. In WT animals, there were no significant differences in primordial follicle numbers across treatment groups (**Figure 3C**). In *Brca1* cKO animals, primordial follicle numbers were unchanged following cyclophosphamide or olaparib treatments alone compared to vehicle control (**Figure 3C**). However, a significant depletion of healthy primordial follicles was observed in mice treated with combination of olaparib and cyclophosphamide (531 ± 45 vs. 311 ± 23, *p* = 0.0456) (**Figure 3C**). The number of abnormal primordial follicles was unchanged between treatment groups and genotypes (**Figure 3D**). No significant differences were observed in the number of healthy or abnormal primary follicles between treatment groups within each genotype (**Figures 3E, F**).

Clinically, serum AMH levels are widely used as a marker for ovarian reserve, despite the fact that AMH is secreted by the growing follicle pool, not primordial follicles (33). No significant difference in AMH levels were observed between treatment groups in both WT and *Brca1* cKO animals (**Figure 3G**). Next, AMH levels were correlated with number of primordial follicles. No significant differences were identified in either WT (**Figure 3H**) or *Brca1* cKO (**Figure 3I**) after control or single therapy cyclophosphamide or olaparib treatment. Primordial follicle number and serum AMH were positively correlated in both WT (r = 0.9429, p = 0.0060, **Figure 3H**) and *Brca1* cKO (r = 0.9276, p = 0.0078, **Figure 3I**) animals that received the combination olaparib cyclophosphamide therapy.

## Discussion

The objective of this study was to investigate the effect of combination olaparib and cyclophosphamide treatment on ovarian function in a conditional knockout mouse model lacking *Brca1* in oocytes. Both WT and *Brca1* cKO mice produced healthy mature oocytes that could undergo fertilization and develop into healthy blastocysts ready for implantation. Similarly, estrous cycling was unchanged between genotypes and treatment groups. Together, this indicates that combination treatment does not impact growing ovarian follicles, hormone dependent cycling, or ovulation and thus, does not likely exert immediate impacts to female reproductive function. However, *Brca1* cKO animals treated with combination therapy had significantly fewer primordial follicles than saline controls, whereas there was no significant depletion in follicles between these groups in WT animals. Primordial follicles form the ovarian reserve representing the entire pool of oocytes that underpin female reproductive lifespan. This observation suggests that loss of BRCA1 in oocytes may increase susceptibility to cyclophosphamide-olaparib-induced reductions in ovarian reserve, potentially contributing to accelerated ovarian aging and a higher risk of long-term reproductive consequences, including diminished fertility and premature ovarian insufficiency (POI).

POI is characterized by the loss of normal ovarian function before the age of 40 and affects approximately 1% of women of reproductive age (34). The etiology of POI is multifactorial, encompassing inherited genetic mutations, environmental exposures, autoimmune disorders, and gonadotoxic cancer therapies (12, 35). Ovarian damage and the risk of POI from cancer treatments are well documented (36, 37). Among these, the detrimental effects of chemotherapy on ovarian function have been most extensively studied (38–41). Chemotherapeutic agents can directly induce DNA damage within oocytes of primordial follicles and accelerate premature follicle depletion by triggering aberrant primordial follicle activation and subsequent “burn-out” of primordial follicles (reviewed in (40)). Collectively, these mechanisms lead to irreversible depletion of the ovarian reserve and increased risk of POI. Importantly, the consequences of POI extend far beyond infertility; premature menopause is associated with elevated risks of cardiovascular disease, osteoporosis, cognitive decline, and adverse mental health outcomes, underscoring the substantial long-term health burden experienced by cancer survivors (42).

Analogous to the menstrual cycle in humans, the estrous cycle in mice is categorized into four-distinct stages with a regular pattern that is associated with ovarian steroid hormone production (43). Monitoring estrous cyclicity therefore provides a sensitive indicator of temporal disruptions to reproductive endocrine function following cancer treatment. Previous studies have demonstrated cyclophosphamide-induced disruption of estrous cyclicity, including loss of estrus and prolonged diestrus, in a range of rodent models and dosing regimens (44–47). However, the magnitude and nature of these effects vary depending on strain, dose, and treatment duration, with some studies reporting altered stage distribution without changes in total cycle length. In the present study, no significant differences were observed in the duration of time spent in each estrous stage between treatment groups or genotypes. This may reflect differences in experimental design, including mouse strain, dosing regimen, and overall treatment exposure, as well as the relatively low cyclophosphamide dose used to compare with studies reporting overt cycle disruption. Collectively, these findings suggest that under the current treatment conditions, estrous cyclicity is relatively resilient despite evidence of underlying ovarian effects on the ovarian reserve. In the context of hormone function, while a limitation of this study is that reproductive hormone profiling beyond AMH was not performed, future studies incorporating stage-matched endocrine analyses may reveal subtle hormonal changes not detected by estrous cyclicity or AMH measurements.

Consistent with the preservation of estrous cyclicity during treatment, IVF outcomes demonstrated that both WT and *Brca1* cKO mice retained responsiveness to gonadotrophin stimulation following treatment. Combination cyclophosphamide-olaparib therapy did not adversely affect oocyte yield, oocyte morphology, fertilization efficiency, or embryo developmental competence, with comparable rates of embryo formation observed across treatment groups and genotypes. PARP1 is constitutively expressed in oocytes during prophase I of meiosis and displays dynamic localization patterns that reflect chromatin organization during oocyte maturation, including germinal vesicle and metaphase II stages (48). This pattern is consistent with proposed roles for PARP1 in regulating chromatin stability and oocyte maturation. In contrast to our findings, a previous study reported that olaparib monotherapy reduced oocyte yield and fertilization rates; however, these discrepancies likely reflect differences in experimental design, including the use of prepubertal mice (PN21) and a substantially higher olaparib dose (300 mg/kg), compared with the adult model and dosing regimen used in the present study (49).

The gonadotoxic effects of cyclophosphamide are well established, with evidence demonstrating both direct DNA damage to oocytes and subsequent loss of primordial follicles (50). In mice, γH2AX immunofluorescence has been detected in primordial follicle oocytes as early as 8 hours following high-dose cyclophosphamide exposure (300 mg/kg), indicating rapid induction of DNA double-strand breaks within the ovarian reserve and apoptosis (51). However, downstream cellular responses may be context or dose dependent. Some studies report limited or delayed apoptosis within primordial follicles, alongside increased activation of growing follicles and upregulation of PI3K/AKT pathway signaling components, suggesting that follicle “burnout” via premature activation may also contribute to follicle depletion (50, 52–54). Interpretation of these findings is complicated by differences in dosing regimens, timing of analysis, and follicle classification strategies across studies, including the grouping of follicle stages in some analyses. Together, these factors contribute to variability in reported outcomes and mechanisms. As such, while cyclophosphamide clearly compromises ovarian reserve, the relative contribution of direct oocyte apoptosis versus accelerated follicle activation remains incompletely resolved.

PARP1 is a nuclear enzyme that catalyses poly(ADP-ribosyl)ation (PARylation), a post-translational modification of proteins that regulates DNA replication and transcription and is essential for maintaining genomic stability (55). Beyond its function in DNA repair, PARP1 also modulates chromatin structure and accessibility through PARylation-dependent interactions with histones and chromatin-associated proteins (56). These functions are particularly relevant during oogenesis and folliculogenesis, which involve extensive and highly regulated chromatin remodeling associated with meiotic progression and follicular development (57, 58). The expression of PARP1 has been localized in the nuclei of oocytes throughout the folliculogenesis from primordial to preantral follicle stage, as well as in granulosa cells of developing follicles (59). Both *in vitro* ovary culture and *in vivo* treatment of olaparib showed that olaparib induced a significant decrease in the number of follicles across all classes in mice (49). This differs from our findings and is likely attributable to key differences in experimental design, including the use of prepubertal animals (PN21), a substantially higher olaparib dose (300 mg/kg), oral administration, and a prolonged treatment duration (2 weeks) in the previous study (49). These factors may influence both drug exposure and ovarian sensitivity, potentially accounting for the divergent outcomes observed.

Following DNA damage, PARP1 is rapidly recruited to sites of DNA lesions, where it facilitates repair signaling through poly(ADP-ribosyl)ation. During apoptosis, PARP1 can be cleaved by cysteine-aspartate proteases (caspases), and detection of cleaved PARP1 is widely used as an early marker of apoptosis (60). Immunofluorescence staining for cleaved PARP1 has been observed in oocytes within primordial follicles of PN7 mice 16 hours after cyclophosphamide treatment (50), consistent with activation of DNA damage and apoptotic pathways in the ovarian reserve. A previous study examining combined cyclophosphamide and olaparib exposure in adult C57BL/6 mice reported no additional reduction in primordial follicle number with combination treatment compared with cyclophosphamide alone (61). However, the comparison was limited to the comparison between cyclophosphamide and cyclophosphamide + olaparib groups.

More recently, Kaseki et al. investigated ovarian toxicity using a global heterozygous Brca1^L63X/+ rat model. and a treatment regimen consisting of a single dose of cyclophosphamide (75 mg/kg) followed by 2 weeks of daily olaparib (25 mg/kg, subcutaneous) (62). However, the timing of tissue collection relative to treatment was not clearly defined. In that study, olaparib monotherapy reduced primordial follicle numbers, whereas combination treatment did not cause additional depletion in either genotype (62). In contrast, neither monotherapy significantly reduced primordial follicles in our study, but combination treatment selectively depleted the ovarian reserve in mice with oocyte-specific Brca1 loss. Kaseki et al. also reported increased granulosa-cell oxidative stress, increased mTOR signaling and reduced PTEN signaling, suggesting that somatic-cell dysfunction and dysregulated follicle activation contribute to ovarian reserve depletion (62). In contrast, our oocyte-specific model isolates the role of BRCA1-dependent DNA repair within oocytes, supporting a mechanism whereby cyclophosphamide-induced DNA crosslinks and PARP inhibition generate cumulative DNA damage that exceeds the repair capacity of BRCA1-deficient oocytes. Thus, in addition to differences in species, mutation model, treatment regimen and tissue collection timing, the divergent findings likely reflect differences in the relative contributions of oocyte-intrinsic DNA repair versus granulosa-cell dysfunction. Future studies incorporating cell type specific conditional knockouts, particularly in granulosa cells, which are known targets of both cyclophosphamide and PARP inhibition, will be important to further delineate their roles in supporting follicle survival and ovarian function.

## Conclusions

This study investigated whether oocyte-specific loss of *Brca1* exacerbates primordial follicle depletion following combined cyclophosphamide and olaparib treatment in mice. Reassuringly, estrous cyclicity and IVF outcomes were unaffected by combination therapy, indicating preserved short-term reproductive function. However, these outcomes do not exclude longer-term effects, as *Brca1* cKO mice exhibited a significant reduction in primordial follicle number following combined treatment, consistent with diminished ovarian reserve. *BRCA1* mutation carriers have previously been reported to have reduced ovarian reserve and an increased risk of POI. Our findings suggest that cyclophosphamide and PARP inhibitor combination therapy may further exacerbate this underlying susceptibility, potentially accelerating ovarian reserve depletion and contributing to adverse long-term reproductive outcomes, including infertility and earlier reproductive ageing. Overall, these data indicate that BRCA1-deficient ovarian tissue may be more vulnerable to combined cyclophosphamide and olaparib exposure, supporting the importance of considering fertility preservation in *BRCA1* variant carriers prior to treatment. However, further studies in clinically relevant models and patient cohorts are required to confirm these findings and establish their clinical significance.

## Data Availability

The original contributions presented in the study are included in the article/**Supplementary Material**. Further inquiries can be directed to the corresponding authors.
