# Supplementary material for: Combination olaparib and cyclophosphamide cancer therapy exacerbates ovarian reserve depletion in mice with conditional loss of Brca1 in oocytes
Source: Front Endocrinol (Lausanne). 2026 Aug 12;17:1885969. doi: 10.3389/fendo.2026.1885969 (PMC13506483; doi:10.3389/fendo.2026.1885969)
Supplement: Supplementary file 1 [file DataSheet1.docx]

**Supplementary Figure**

**Supplementary Figure 1.** The effects of combination treatment of cyclophosphamide and olaparib on body weight in WT and Brca1 cKO PN40-80 mice. Animal body weight at collection (**A**). Each data point represents one animal (Control WT n=6; Control cKO n=6; Cyclo WT n=6; Cyclo cKO n=6; Olap WT n=6; Olap cKO n=6; Cyclo+Olap WT n=6; Cyclo+Olap cKO n=6;). Data are presented as mean ± SEM. One-way ANOVA with Holm-Šídák post-hoc test for parametric data, Kruskal-Wallis test with Dunn’s multiple comparison post-hoc test for non-parametric data. Data are considered significant at p<0.05.
